# ROS-induced allosteric modulation of NikR promotes *Helicobacter pylori* biofilm formation by attenuating FlgR-dependent inhibition of the molybdate transport system

**DOI:** 10.1080/21505594.2025.2589562

**Published:** 2025-11-12

**Authors:** Yantong Zheng, Shutong Li, Junyuan Xue, Lu Zhang, Liyuan Wang, Yican Zhao, Wenxin Zhang, Wenyue Ma, Jinmeng Liu, Yanlin Sun, Yundong Sun

**Affiliations:** a Key Laboratory for Experimental Teratology of the Ministry of Education and Department of Microbiology, School of Basic Medical Sciences, Shandong University, Jinan, Shandong, China; b Section of Infection and Immunity, Herman Ostrow School of Dentistry, Norris Comprehensive Cancer Center, University of Southern California, Los Angeles, CA, USA; c Department of Pathology, School of Basic Medical Sciences, Cheeloo College of Medicine, Shandong University, Jinan, Shandong, China

**Keywords:** *Helicobacter pylori*, ROS, biofilms, FlgR, molybdate transport system, NikR

## Abstract

*Helicobacter pylori* biofilm formation is crucial for its persistence and transmission, constituting a notable public health concern. Understanding the regulatory mechanisms driving biofilm initiation is vital for developing effective control strategies. This study reveals a previously uncharacterized regulatory mechanism where reactive oxygen species (ROS) promote *H. pylori* biofilm formation by modulating the key flagellar regulator FlgR and the molybdate transport system ModABD. We demonstrate that FlgR acts as a repressor of biofilm development. Mechanistically, FlgR inhibits the transcription of the *modABD* operon, essential for biofilm formation, by suppressing the activity of sigma factor σ^28^. Crucially, we identify the nickel-responsive regulator NikR as a repressor of *flgR* expression. ROS induces a conformational change in NikR, converting it to its DNA-binding holo-form, which directly binds the *flgR* promoter and represses its expression. This repression alleviates FlgR-mediated inhibition of σ^28^, thereby de-repressing the *modABD* operon and facilitating the transition from planktonic to biofilm growth. Our findings uncover a previously unknown ROS-NikR-FlgR-σ^28^-ModABD signaling axis governing *H. pylori* biofilm formation.

## Introduction

*Helicobacter pylori* (*H. pylori*) is a microaerophilic, gram-negative, spiral-shaped bacterium that persistently colonizes the human gastric mucosa [1]. It is closely associated with various gastrointestinal diseases, including chronic gastritis, peptic ulcers, and gastric cancer [2], with the World Health Organization (WHO) classifying it as a Group 1 carcinogen [3]. More than 50% of the global population is infected with *H. pylori*, highlighting its significant impact on public health worldwide [4]. Transmission primarily occurs via fecal-oral, oral-oral, and gastro-oral routes [5,6]. Crucially, as a microaerophilic pathogen, *H. pylori* inevitably encounters oxygen stress both during transmission (e.g. atmospheric oxygen) [7] and within the host environment (e.g. phagocyte-derived ROS) [8]. Notably, biofilm formation significantly enhances *H. pylori* survival in these diverse and hostile environments, thereby complicating the eradication efforts [9–12]. It serves as a critical defense mechanism, protecting the bacterium against the pervasive oxygen stress encountered both in transit and during infection [13,14], while also conferring increased resistance to antimicrobial agents [15].

Biofilms are bacterial co-aggregates surrounded by extracellular polymeric substances (EPS), which are primarily composed of polysaccharides, proteins, and extracellular DNA (eDNA) [16]. These components mediate bacterial adhesion to both abiotic and biotic surfaces and provide a protective barrier against environmental stressors [17], contributing to the development of various chronic clinical infections [18,19]. Additionally, the metabolic heterogeneity within biofilms activates stress response pathways (e.g. oxidative, stringent, and SOS), which enhances bacterial adaptation and accelerates the evolution of drug resistance [20,21]. Therefore, elucidating the molecular mechanisms that govern *H. pylori* biofilm formation and regulation is essential for preventing transmission and developing effective strategies to control *H. pylori* infection.

Bacterial biofilm formation typically involves four stages: initial attachment, microcolony formation, maturation, and dispersion [22]. In *H. pylori*, adhesion proteins (AlpB, OipA, SabA, NapA) and chemotaxis components (CheA, CheW) mediate surface attachment [23–25], and quorum sensing (AI-2, DSF) regulates aggregation or dispersion [26]. While various factors contributing to *H. pylori* biofilm formation have been identified, the regulatory mechanisms underlying its transition from planktonic to sessile states, particularly in response to the omnipresent oxygen stress, remain poorly understood.

Motility is a key determinant of biofilm initiation [27]. As the primary motility organelle, flagella facilitate surface attachment, while their subsequent repression stabilizes biofilm growth [27–29]. A similar mechanism has been described in *Pseudomonas aeruginosa*, where surface sensing triggers cyclic di-GMP (c-di-GMP) accumulation, repressing the flagellar gene *fliC* to promote biofilm initiation [30]. This suggests that motility suppression may represent a conserved strategy in *H. pylori* biofilm formation.

In *H. pylori*, flagellar assembly is hierarchically regulated by σ^80^ (class 1), σ^54^ (class 2), and σ^28^ (class 3) [31]. FlgR, a σ^54^ enhancer-binding protein, activates class 2 genes (rod and hook) while repressing σ^28^-dependent class 3 genes (e.g. *flaA*) [32–35]. Although FlgR is essential for motility and host colonization [32], its role in biofilm regulation has not yet been elucidated.

In this study, we demonstrate that the key flagellar regulator FlgR represses biofilm formation through inhibition of the molybdate transporter system ModABD. We further identify that ROS induces allosteric modulation of the nickel-responsive regulator NikR, which in turn represses *flgR* expression and promotes biofilm formation. Our findings reveal novel insights into the regulatory axis controlling *H. pylori* biofilm initiation and offer a new perspective for developing targeted anti-biofilm strategies to prevent its colonization and transmission.

## Materials and methods

### Bacterial strains and culture conditions

*H. pylori* strains 26695, G27, and H57 were obtained from Dr. Jianzhong Zhang (Chinese Center for Disease Control and Prevention), Dr. Hongkai Bi (Nanjing Medical University), and Dr. Zhenghong Chen (Guizhou Medical University), respectively. These strains were cultured on Mueller-Hinton agar (Oxoid, CM0337) supplemented with 5% (v/v) defibrinated sheep blood or in Brucella broth (Haibo Biotechnology, HB0241) containing 10% (v/v) newborn calf serum. Cultures were incubated microaerobically (85% N_2_, 10% CO_2_, and 5% O_2_) at 37°C with shaking at 120 rpm. *Escherichia coli* (*E. coli*) TOP10 and BL21(DE3) strains were cultured in Luria-Bertani (LB) broth (Haibo Biotechnology, HB0128) or on LB agar (Haibo Biotechnology, HB0129) at 37°C with shaking at 200 rpm.

The clinical *H. pylori* strain H57 used in this study was generously provided by Professor Zhenghong Chen of Guizhou Medical University isolated from gastric biopsy samples of a refractory peptic ulcer patient during routine diagnostic endoscopy following multiple failed antibiotic treatments. Verbal informed consent was obtained from all participating patients prior to sample collection. This consent procedure was explicitly approved by the Guizhou Medical University Ethics Committee (Ethics review approval number: 2016–56). The rationale for verbal (rather than written) consent was based on the following considerations: 1. Therapeutic Purpose Alignment: Bacterial isolation and antimicrobial susceptibility testing were performed exclusively for the patient’s own clinical management, aiming to identify effective eradication regimens tailored to their specific infection. 2. Minimal Risk and Routine Procedure: The isolation process posed no additional risks beyond standard diagnostic endoscopy, as samples were obtained solely from residual tissue collected during clinically indicated procedures. No extra interventions were performed. 3. Immediate Clinical Benefit: Results directly guided the patient’s subsequent treatment, constituting an integral extension of standard-of-care practices for treatment-resistant infections. 4. Privacy Protection: Verbal consent avoided generating identifiable documents for a procedure indistinguishable from routine diagnostics. Patients received a comprehensive verbal explanation regarding: The purpose of bacterial culture and susceptibility testing; the use of residual biological material exclusively for their personalized treatment; anonymized data handling in potential future research; and their right to decline without affecting ongoing care. Consent discussions were witnessed by an independent clinical staff member and documented in the medical record. Retrospective research using de-identified bacterial isolates was covered by a separate written consent waiver granted by the Institutional Review Board of the School of Basic Medical Sciences, Shandong University (Protocol No. ECSBMSSDU2020-1–021), as the study exclusively involved anonymized microbiological analysis with no patient data linkage.

### Construction of H. pylori mutant and complemented strains

Deletion mutants of *flgR*, *modA*, and *nikR* were generated in *H. pylori* strains 26695, G27, and H57 via allelic exchange. Target genes were replaced by a kanamycin resistance cassette (*aphA-3*) using homologous recombination, as previously described [36]. Complemented strains (*flgR*^C^, *modA*^C^) were constructed by chromosomal insertion of the respective intact gene with a chloramphenicol resistance marker between loci *hp0203* and *hp0204* in the 26695 Δ*flgR* or Δ*modA* background. The schematic maps of mutant and complemented strains are provided in Figure S1. All mutants and complemented strains were verified by PCR and Sanger sequencing. Primer sequences are listed in Table S1.

### Growth curves

*H. pylori* 26695 WT, Δ*flgR* and Δ*modA* strains were cultured overnight at 37°C under microaerobic conditions (85% N_2_, 10% CO_2_, and 5% O_2_) with shaking at 120 rpm. Cultures were adjusted to an initial OD_600_ of 0.08 with fresh liquid medium. Bacterial growth was monitored every 12 h over 144 h by OD_600_ measurements. Three biological replicates were performed for each strain.

### Biofilm formation assay

Biofilms were cultured using the “colony biofilm” method [37]. To simulate the oxidative pressure encountered by *H. pylori* during transmission and host infection, hydrogen peroxide (H_2_O_2_) was incorporated into the biofilm induction system. Briefly, *H. pylori* cultures in the logarithmic growth phase were diluted to 1 × 10^8^ CFU/mL. A 25 μL aliquot of the bacterial suspension was deposited onto solid Mueller–Hinton agar supplemented with 5% defibrinated sheep blood and 50 μM H_2_O_2_, then overlaid with a sterile nitrocellulose membrane (Millipore, HATF00010). The plates were incubated in an inverted position at 37°C under microaerobic conditions (85% N_2_, 10% CO_2_, and 5% O_2_) for 3 days to promote biofilm development. Biofilm morphology was documented using a Zeiss stereo microscope (Zeiss, 435,003-9901–000) equipped with an Axiocam 506 color camera.

### Confocal laser scanning microscopy (CLSM)

Biofilms were gently washed with phosphate-buffered saline (PBS) to remove planktonic cells. Samples were stained with one of the following: Viability: SYTO 9 and propidium iodide (PI) from the LIVE/DEAD BacLight kit (Thermo Fisher, L13152) for 30 min in the dark. Polysaccharides: Calcofluor White (Sigma, BCCF8014) for 15 min. Proteins: SYPRO Ruby biofilm matrix stain (Thermo Fisher, 2,399,418) for 30 min. Excess dye was removed by PBS washes. Images were acquired using a Zeiss confocal microscope (63× oil immersion objective; Zeiss, LSM880) with Z-stack scanning. 3D reconstructions were generated using ZEN 3.3 software. Quantitative analysis of biofilm biomass and fluorescence intensity were performed based on CLSM images using COMSTAT plugin [38] and ImageJ software, respectively.

### Scanning electron microscopy (SEM)

Biofilms were fixed in 2.5% (v/v) glutaraldehyde at 4°C overnight, washed thrice in 0.1 M phosphate buffer (PB; pH 7.4), post-fixed with 1% (w/v) osmium tetroxide for 2 h, and rinsed again in PB. Samples were dehydrated in an ethanol series (30–100%), critical-point dried, sputter-coated with gold, and imaged using a Hitachi SEM (Hitachi, SU8100).

### Biofilm biomass quantification

Crystal violet staining was performed as follows: biofilms were washed with PBS, detached from membranes in 1 mL PBS, and stained with 0.1% (w/v) crystal violet for 5 min. Unbound dye was removed by triple PBS washing. Bound dye was solubilized in 1 mL of absolute ethanol, and absorbance was measured at 595 nm (Agilent, SH1M2F Multimode Reader).

### RNA sequencing and analysis

*H. pylori* 26695 WT and Δ*flgR* strains were cultured to the logarithmic growth phase, collected, and diluted to an OD_600_ of 1.0 with PBS. The cells were then treated with 50 μM H_2_O_2_ and cultured at 37°C for 1 h under microaerobic conditions. Cells were harvested by centrifugation at 6000 rpm for 5 min at 4°C, washed twice with PBS, snap-frozen in liquid nitrogen for 5–10 min, and stored at −80°C. RNA sequencing was performed by Majorbio Bio-pharm Technology Co., Ltd. on the NovaSeq X Plus platform with a read length of 150 bp.

### RNA extraction and quantitative real-time pcr (qRT-PCR)

Total RNA was extracted using RNAex Pro Reagent (Accurate Biotechnology, AG21102), treated with DNase I, and reverse-transcribed using Evo M-MLV RT Mix (Accurate Biotechnology, AG11728). qRT-PCR was performed with SYBR Green Premix Pro Taq HS (Accurate Biotechnology, AG11701) on a QuantStudio 3 system (Thermo Fisher, 272,327,021). *16S rRNA* served as the endogenous control. Relative expression was calculated via the 2^−ΔΔCT^ method. Conventional RT-PCR products were analyzed by 1.5% agarose gel electrophoresis. Primers are listed in Table S1.

### Expression and purification of σ^28^ and NikR recombinant proteins

Expression of σ^28^ and NikR proteins was conducted as described previously [39]. Briefly, DNA fragments encoding σ^28^ and NikR, excluding the terminator sequences from the *H. pylori* 26695 genome, were cloned into the pET-32a plasmid by homologous recombination. The constructs were transformed into *E. coli* BL21(DE3) and grown overnight in LB medium at 37°C. When the culture reached an OD_600_ of 0.6, protein expression was induced with 0.5 mM isopropyl β-D-1-thiogalactopyranoside (IPTG). For σ^28^, induction was performed after 20 h of shaking at 16°C, while NikR expression was induced after 4 h of shaking at 37°C. The proteins were purified by affinity chromatography using Ni-IDA Sepharose Resin (Sangon Biotech, C600029) and eluted with stepwise concentrations of imidazole. Protein purity was assessed by 10% SDS-PAGE and Coomassie Brilliant Blue staining (Servicebio, G2059).

### Luciferase reporter assay

Promoter regions of *modA* and *flgR* were cloned into pGL3-Basic (Tsingke, BR014) to generate pGL3-OP*_modA_* and pGL3-OP*_flgR_*. Plasmids were co-transformed into *E. coli* BL21(DE3) with pET32a-σ^28^ or pET32a-NikR. Luciferase activity was measured using a Luciferase Assay Kit (Yeasen, 11401ES60) and Centro XS3 luminometer (Berthold, LB960). Controls included empty pGL3-Basic and pET32a vectors.

### Electrophoretic mobility shift assay (EMSA)

The EMSA was performed as previously described [15]. Briefly, DNA probes were amplified from *H. pylori* 26695 genomic DNA (Table S1). Purified σ^28^ or NikR protein was incubated with probes in the EMSA binding buffer (Beyotime, GS005) for 30 min at 25°C. Complexes were resolved on 6% non-denaturing polyacrylamide gels in 0.25× TBE at 150 V for 85 min. Gels were stained with GelRed (Yeasen, 10203ES76) and visualized using a UV transilluminator (Tanon, 2500).

### In vitro experimental evolution assay

Based on previous methods with minor modifications [40,41], evolution was initiated using the *H. pylori* Δ*modABD* strain as the ancestral background. Logarithmic-phase cultures (1 × 10^8^ CFU/mL) were inoculated onto a solid medium containing 50 μM H_2_O_2_ and passaged serially 15 times. After washing with PBS, cultures were adjusted to 10^6^ CFU/mL, and 100 μL aliquots were plated onto 10 individual plates containing 200 μM H_2_O_2_. Plates were incubated at 37°C under microaerobic conditions for 2 days. From each plate, a single colony was isolated and further passaged five times on medium containing 50 μM H_2_O_2_, and biofilm formation comparable to that of the wild-type strain was confirmed. Ten successfully adapted clones were stored at −80°C for downstream analysis. Whole-genome sequencing was performed on these evolved strains (M1–M10), and single nucleotide polymorphisms (SNPs) were subsequently identified.

### Whole-genome sequencing and SNP identification

We extracted genomic DNA from the M1–M10 strains using the TIANamp Bacteria DNA Kit (Tiangen, DP302). DNA concentration and quality were determined using a Spectrophotometer (Thermo, NanoDrop2000). Libraries for Illumina sequencing were constructed for each accession according to the manufacturer’s specifications. After DNA library construction, whole-genome sequencing was performed by Biomarker Technologies using the Illumina NovaSeq platform (paired-end 150 bp). Raw reads were processed with fastp to remove adapters, low-quality reads, and reads containing poly-N. The clean reads were then aligned to the *H. pylori* 26695 reference genome (NCBI accession no. GCF_000008525.1) using BWA-MEM2. Variants were called with GATK HaplotypeCaller v3.8 and filtered to retain high-confidence SNPs and InDels based on read depth and quality. Annotation was performed with SnpEff v3.6c [42], with coding SNPs classified as synonymous or nonsynonymous, and InDels in coding regions evaluated for frameshift effects.

### Statistical analysis

All experiments were performed in at least three independent replicates, and data were presented as mean ± SD. Differences were analyzed by unpaired Student’s *t*-test (two groups) or one-way ANOVA with Tukey’s post-hoc test (≥3 groups) using GraphPad Prism 8.0.2. Significance was defined as *p* < 0.05.

## Results

### Reduced FlgR expression enhances H. pylori biofilm formation

To assess the involvement of flagellar genes in *H. pylori* biofilm development, we analyzed transcriptomic data from our previous study comparing planktonic and biofilm-associated cells [25]. This analysis revealed significant downregulation of genes encoding flagellar structural components (hook, filament, basal body) and the master regulator *flgR* in biofilm cells (Figure S2), suggesting coordinated transcriptional repression potentially mediated by FlgR.

**Figure 1.**
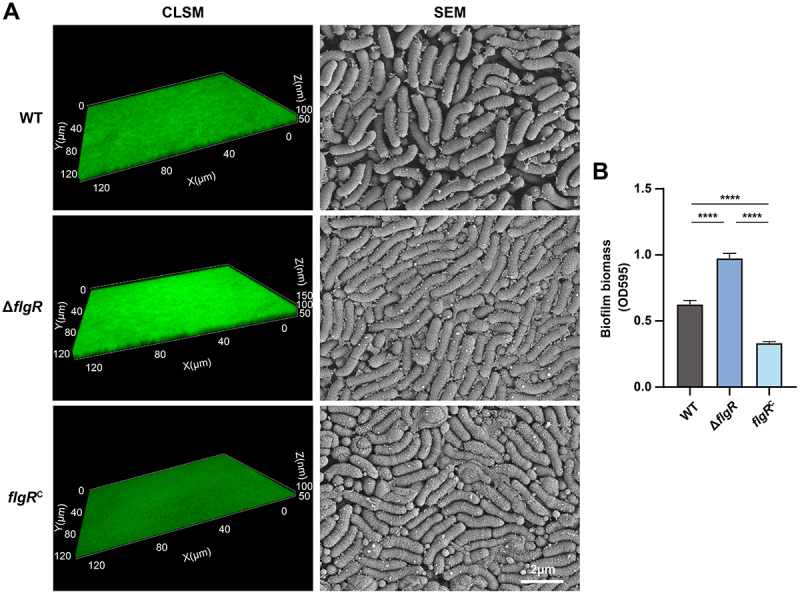
FlgR deficiency enhances biofilm formation in *H. pylori* 26695. (A) Biofilm architecture of WT, Δ*flgR*, and *flgR*^C^ strains. Left: CLSM images of SYTO 9-stained biofilms formed on medium containing 50 μM H_2_O_2_ after 72 h microaerobic incubation. Right: SEM images showing structural details at 30,000× magnification. (B) Quantitative biofilm biomass analysis by crystal violet staining. Data represent the mean ± SD from triplicate measurements. ****p < 0.0001.

To investigate FlgR’s role, we generated a *flgR* deletion mutant (Δ*flgR*) and its complemented strain (*flgR*^*C*^) in *H. pylori* 26695. While *flgR* deletion did not affect bacterial growth (Figure S3), it profoundly altered biofilm architecture. CLSM showed that Δ*flgR* biofilms were markedly thicker and more compact than wild-type (WT, *H. pylori* 26695) or *flgR*^C^ biofilms (Figure 1(A)). SEM further confirmed denser cellular packing in Δ*flgR* biofilms (Figure 1(A)). Quantification of biofilm biomass by crystal violet staining corroborated these observations, demonstrating a significant increase in biomass for the Δ*flgR* strain compared to WT and *flgR*^C^ (Figure 1(B)). The slight difference in biofilm formation between WT and *flgR*^C^ likely arises from local chromosomal context effects or subtle regulatory variations rather than incomplete complementation. Quantitative analysis of biofilm biomass using COMSTAT confirmed that although *flgR*^C^ exhibited a slightly reduced biofilm volume compared with WT, the difference was not statistically significant (Figure S4), supporting the functional restoration of FlgR. These results establish FlgR as a negative regulator of *H. pylori* biofilm formation.

### The molybdate transporter ModABD is essential for robust biofilm formation

To identify FlgR-regulated pathways, we performed RNA sequencing on WT and Δ*flgR* strains treated with 50 µM H_2_O_2_ for 1 h. We identified 182 differentially expressed genes (DEGs) in Δ*flgR* versus WT (168 upregulated, 14 downregulated; Figure S5A). KEGG pathway analysis highlighted DEGs associated with “bacterial motility,” “membrane transport,” and “glycan biosynthesis and metabolism” (Figure S5B). Gene Ontology (GO) enrichment analysis indicated significant upregulation of genes encoding integral membrane components (Figure S5C). Notably, all structural genes (*modA* [*hp0473*], *modB* [*hp0474*], *modD* [*hp0475*]) of the molybdate transport operon (*modABD*) were upregulated in Δ*flgR* (Figure S5A). Validation by qRT-PCR confirmed significant upregulation of *modA*, *modB*, and *modD* expression in Δ*flgR* under both planktonic and biofilm conditions compared to WT and *flgR*^C^ (Figure S5D), indicating FlgR represses the molybdate transport system.

Given the *modABD* operon’s upregulation in Δ*flgR* and its role as an ATP-binding cassette (ABC) type transporter for high-affinity molybdate uptake [43–45], we investigated its contribution to biofilm formation. We constructed a *modA* deletion mutant (Δ*modA*), its complemented strain (*modA*^C^), and additional mutants (Δ*modB*, Δ*modD*, Δ*modABD*) in *H. pylori* 26695. *ModA* deletion did not impact growth (Figure S3).

**Figure 2.**
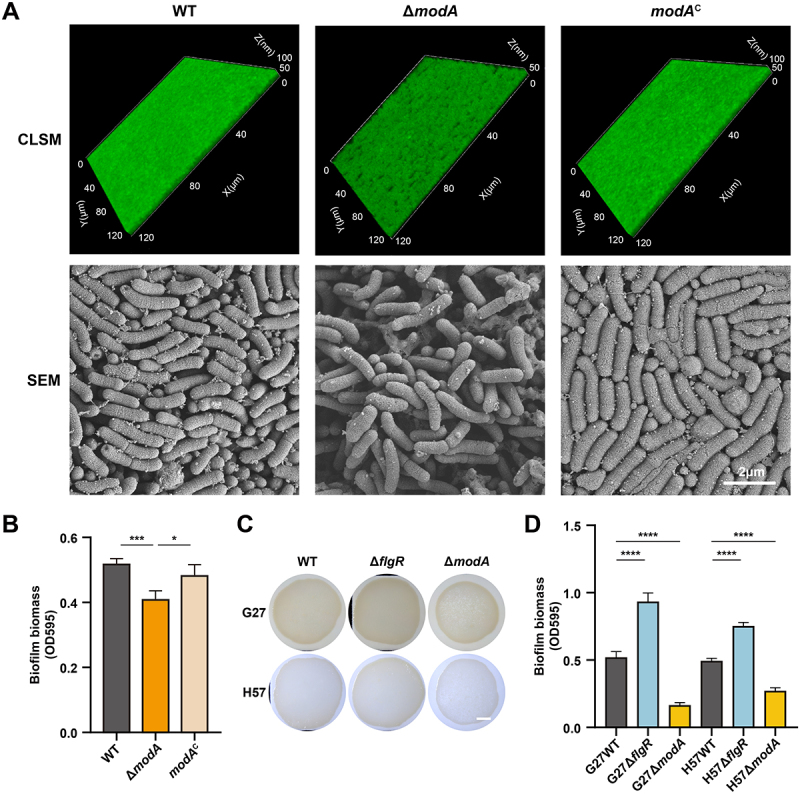
ModABD mediates biofilm formation across *H. pylori* strains. (A-B) biofilm architecture of *H. pylori* 26695 WT, Δ*modA*, and *modA*^C^ strains. Top: CLSM images of SYTO 9-stained biofilms formed on medium containing 50 μM H_2_O_2_ after 72 h; bottom: SEM images showing structural details at 30,000× magnification. Quantified biofilm biomass by crystal violet staining. Data represent the mean ± SD from quadruplicate measurements. *p < 0.05, ***p < 0.001. (C) Biofilms formed by wt, Δ*flgR*, and Δ*modA* strains in *H. pylori* G27 (top) and clinical isolate H57 (bottom) after 72 h of growth on medium containing 50 μM H_2_O_2_ covered with nitrocellulose membranes. Scale bar = 200 μm. (D) Quantified biofilm biomass by crystal violet staining. Data represent the mean ± SD from quadruplicate measurements. ****p < 0.0001.

Characterization of biofilms revealed striking defects upon ModABD disruption. CLSM showed that Δ*modA* biofilms possessed a looser architecture, increased cavities, and significantly reduced thickness compared to WT and *modA*^C^ biofilms (Figure 2(A)). SEM confirmed sparser bacterial arrangements within Δ*modA* biofilms (Figure 2(A)). Crystal violet staining quantified a significant reduction in biofilm biomass for Δ*modA* relative to WT and *modA*^C^ (Figure 2(B)). Similar impairments were observed in Δ*modB*, Δ*modD*, and Δ*modABD* biofilms (Figure S6), COMSTAT-based quantification revealed that Δ*modB*, Δ*modD*, and Δ*modABD* mutants exhibited reduced biofilm biomass compared with WT, with a significant decrease observed in Δ*modABD*, demonstrating that an intact ModABD system is crucial for *H. pylori* biofilm development. To further assess the effect of ModABD on extracellular matrix composition, CLSM analysis revealed that Δ*modA* biofilms exhibited marked reductions in extracellular DNA/RNA (PI), polysaccharides (Calcofluor White), and proteins (SYPRO Ruby) compared with WT under H_2_O_2_ stress (Figure S7), suggesting that ModABD-mediated molybdate transport supports EPS production and biofilm stability.

To assess conservation across strains, we generated Δ*flgR* and Δ*modA* mutants in the standard strain G27 and the clinical antibiotic-resistant isolate H57. Phenotypic analysis showed that Δ*flgR* mutants consistently formed darker, denser biofilms than their respective WT strains in both G27 and H57 backgrounds (Figure 2(C)). Conversely, Δ*modA* biofilms appeared lighter, exhibited central collapse, and displayed visible gaps (Figure 2(C)). Crystal violet quantification confirmed a significantly increased biomass for Δ*flgR* and decreased biomass for Δ*modA* across both strains (Figure 2(D)), supporting conserved roles for FlgR and ModABD in biofilm regulation in diverse *H. pylori* strains.

### FlgR represses ModABD expression by inhibiting σ^28^ activity

FlgR, a σ^54^ enhancer-binding protein, represses σ^28^-dependent class 3 flagellar genes (e.g. *flaA*) [32,33]. As FlgR lacks a canonical DNA-binding domain [35,46], we hypothesized it indirectly regulates *modABD*. Bioinformatics identified a conserved σ^28^ recognition motif (TAAANNNNNNNNNNCGAT) in the *modABD* promoter (Figure S8), suggesting potential σ^28^ regulation similar to *E. coli* [47].

**Figure 3.**
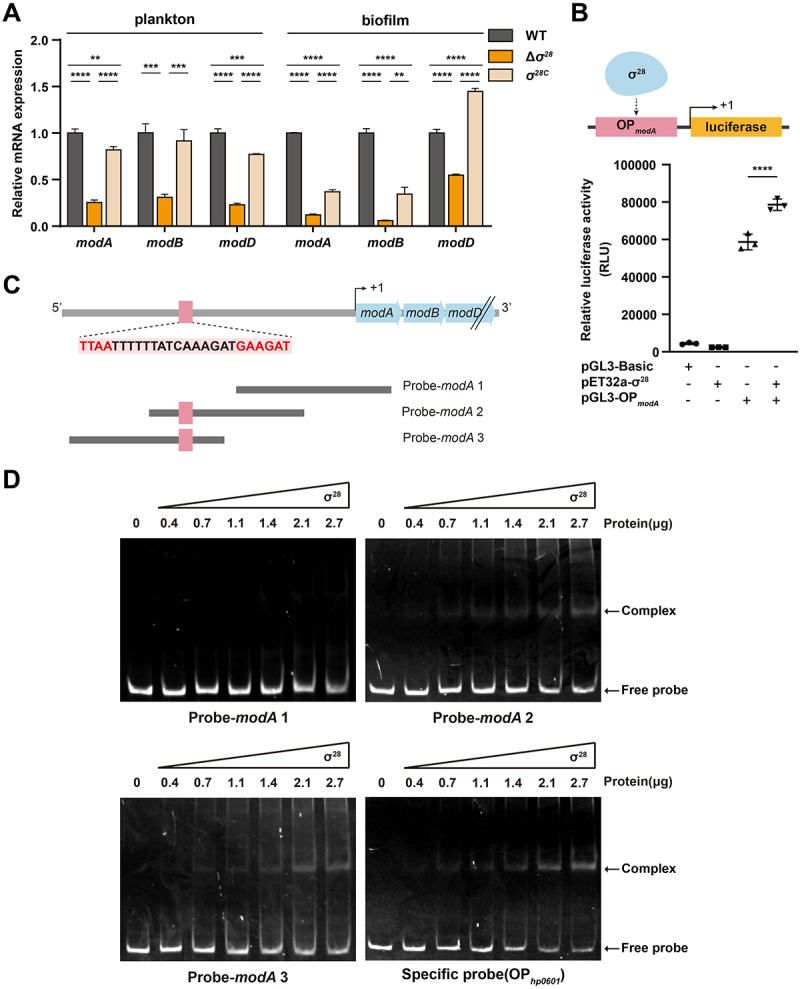
σ^28^ activates molybdate transport system expression in *H. pylori*. (A) Relative mRNA expression levels of *modA*, *modB*, and *modD* in WT, Δ*σ*^*28*^, and *σ*^*28*C^ strains under planktonic and biofilm conditions, using *16S rRNA* as the internal control. Data represent the mean ± SD from triplicate measurements. **p < 0.01, ***p < 0.001, ****p < 0.0001. (B) Top: luciferase reporter assay schematic. Bottom: relative luciferase activity in *E. coli* cells co-transformed with pGL3-Basic, pGL3-OP_*modA*_, and pEt32a-σ^28^ in different combinations. Data represent the mean ± SD from triplicate measurements. ****p < 0.0001. (C) Schematic of the *modA* promoter probes. Red: σ^28^ consensus sequence; black: *H. pylori* 26695 genomic sequence; blue: structural genes in the *modABD* operon. All probes are 150 bp in length. (D) EMSA showing purified σ^28^ protein binding to the *modA* promoter. Probes: probe-*modA* 1, Probe-*modA* 2, Probe-*modA* 3, and OP_*hp0601*_ (positive control). Complex: DNA-protein complexes; free probe: unbound dna probes; protein concentrations: 0, 0.4, 0.7, 1.1, 1.4, 2.1, and 2.7 μg.

qRT-PCR analysis demonstrated that deletion of σ^28^ (Δ*σ^28^*) significantly reduced *modA*, *modB*, and *modD* expression compared to WT and the complemented strain (*σ^28^*^C^) under both planktonic and biofilm conditions (Figure 3(A)), indicating σ^28^ positively regulates *modABD*.

A luciferase reporter assay using the *modA* promoter cloned into pGL3-Basic (pGL3-OP*_modA_*) (Figure 3(B), top) provided direct evidence. Co-expression of σ^28^ (pET32a-σ^28^) significantly increased luciferase activity from pGL3-OP*_modA_* compared to pGL3-OP*_modA_* alone (Figure 3(B), bottom). Controls (pGL3-Basic, pET32a-σ^28^ alone) showed minimal activity.

EMSA confirmed the specific binding. Purified σ^28^ protein formed complexes with DNA probes (Figure 3(C)), Probe-*modA* 2, Probe-*modA* 3, and positive control OP*_hp0601_* [48] containing the σ^28^ consensus motif, resulting in clear mobility shifts (Figure 3(D)). In contrast, Probe-*modA* 1, lacking the motif, showed no shift (Figure 3(D)). These results demonstrate that σ^28^ directly binds the *modABD* promoter and activates transcription. FlgR, by repressing σ^28^ activity, indirectly inhibits *modABD* expression, thereby modulating the planktonic-to-biofilm transition.

### NikR represses flgR expression in response to ROS and nickel

To investigate the mechanism underlying FlgR repression during biofilm formation, we reanalyzed transcriptome sequencing data from *H. pylori* biofilms and visualized the differential expression of transcriptional regulators. As shown in Figure S9, the expression of *hp1338* was significantly reduced in biofilm cells compared with planktonic cells. The *hp1338* gene encodes NikR, a nickel-dependent regulator [49]. NikR responds to fluctuations in intracellular nickel levels by undergoing conformational changes and plays a critical role in global gene regulation [50–52]. Bioinformatics identified a putative NikR binding motif (TRWYA-n15-TRWYA) within the *flgR* operon promoter (Figure S10).

**Figure 4.**
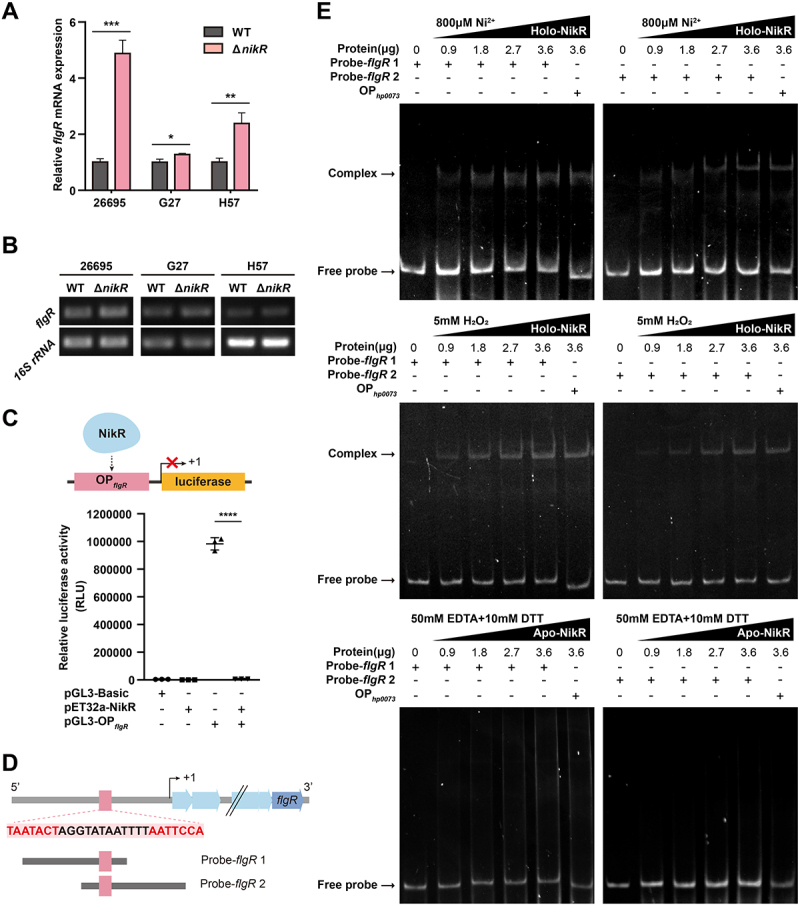
NikR represses *flgR* expression in *H. pylori*. qRT-PCR. (A) and RT-PCR. (B) Analysis of the relative mRNA expression levels of *flgR* in WT and Δ*nikR* strains of *H. pylori* 26695, G27, and H57, using *16S rRNA* as the internal control. Data represent the mean ± SD from triplicate measurements. *p < 0.05, **p < 0.01, ***p < 0.001. (C) Top: luciferase reporter assay schematic. Bottom: relative luciferase activity in *E. coli* cells co-transformed with pGL3-Basic, pGL3-OP_*flgR*_, and pEt32a-NikR in different combinations. Data represent the mean ± SD from triplicate measurements. ****p < 0.0001. (D) Schematic of the *flgR* promoter probes. Red: NikR consensus sequence; black: *H. pylori* 26695 genomic sequence; blue: structural genes in the *flgR* operon. All probes are 120 bp in length. (E) EMSA showing purified NikR binding to the *flgR* promoter under 800 μM Ni^2+^, 5 mM H_2_O_2_, or a combination of 50 mM EDTA and 10 mM DTT conditions. Probes: probe-*flgR* 1, Probe-*flgR* 2, and OP_*hp0073*_ (positive control). Complex: DNA-protein complexes; free probe: unbound DNA probes; protein concentrations: 0, 0.9, 1.8, 2.7, and 3.6 μg.

qRT-PCR and RT-PCR confirmed this regulatory relationship: *flgR* expression was significantly upregulated in Δ*nikR* mutants compared to WT in strains 26695, G27, and H57 (Figure 4(A,B)), demonstrating NikR represses *flgR* across diverse backgrounds.

A luciferase reporter assay using the *flgR* promoter (pGL3-OP*_flgR_*) showed that co-expression of NikR (pET32a-NikR) significantly reduced reporter activity compared to pGL3-OP*_flgR_* alone (Figure 4(C)), indicating NikR binding represses *flgR* promoter activity.

NikR is known to regulate gene expression only upon nickel binding, which triggers its transition from the apo to the holo conformation [52,53]. However, the intracellular nickel levels did not differ significantly between planktonic and biofilm cells [54], suggesting an alternative activation mechanism during biofilm adaptation. Considering that low-concentration H_2_O_2_ effectively induced biofilm formation in our model, we hypothesized that ROS may trigger NikR allosteric activation. EMSA using purified NikR protein and *flgR* promoter probes containing NikR binding motif (Probe-*flgR* 1, Probe-*flgR* 2; Figure 4(D)) elucidated the activation mechanism. The DNA-binding activity of NikR was conformation-dependent. Holo-NikR, formed by incubation with 800 µM Ni^2+^, bound robustly to both probes and the positive control OP*_hp0073_* [53], causing significant mobility shifts (Figure 4(E)). Crucially, the treatment with 5 mM H_2_O_2_ also induced NikR binding, mimicking the effect of nickel. In contrast, apo-NikR (induced by chelators EDTA and DTT) exhibited minimal binding (Figure 4(E)). These results establish that ROS and nickel induce a conformational change in NikR to its DNA-binding holo form, enabling direct repression of *flgR* expression.

### Compensatory evolution restores biofilm formation in the ΔmodABD mutant under oxidative stress

**Figure 5.**
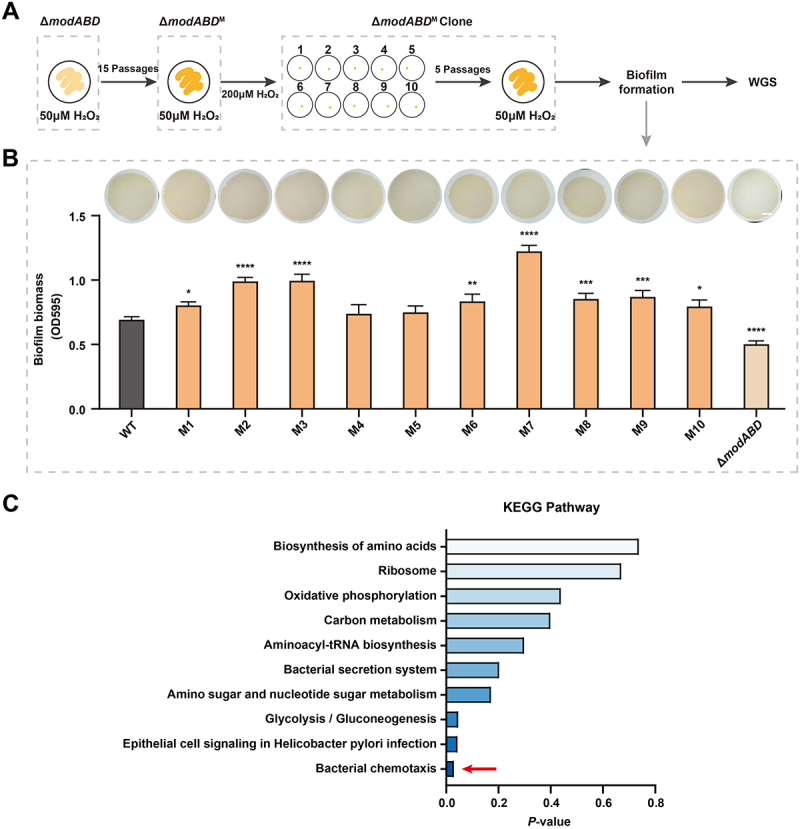
H_2_O_2_-induced adaptive evolution enhances biofilm formation in Δ*modA*BD mutants. (A) Experimental design for oxidative stress adaptation. WGS: whole-genome sequencing. (B) Biofilm formed by the wt strain, the M1–M10 strains, and the non-induced Δ*modABD* strain after 72 h of growth on medium containing 50 μM H_2_O_2_, covered with nitrocellulose membranes (top). Quantitative biofilm biomass analysis by crystal violet staining (bottom). Data represent the mean ± SD from quadruplicate measurements. *p < 0.05, **p < 0.01, ***p < 0.001, ****p < 0.0001. Scale bar = 200 μm. (C) Kegg pathway enrichment of mutated genes in M7. Darker to lighter colors indicate lower to higher *p*-values, as determined by the kolmogorov – Smirnov test.

To further elucidate the role of ModABD in *H. pylori* biofilm formation, we performed an *in vitro* evolution assay with the Δ*modABD* mutant strain under sublethal oxidative stress. The Δ*modABD* strain was serially passaged 15 times on solid medium containing 50 μM H_2_O_2_ (Figure 5(A)). After evolution, we screened the endpoint population and isolated 10 evolved clones (M1–M10) that exhibited markedly enhanced biofilm formation compared with the unevolved Δ*modABD* strain (Figure 5(B)). Quantitative crystal violet assays showed that these clones achieved biofilm biomass comparable to or even exceeding that of the WT strain (Figure 5(B)).

Whole-genome sequencing of all 10 evolved clones revealed recurrent single-nucleotide polymorphisms (SNPs). KEGG enrichment analysis of mutations identified in clone M7 showed significant enrichment in the “bacterial chemotaxis” pathway (*p* = 3.03 × 10^−2^; Figure 5(C)), including a premature stop mutation in *cheA*, a frameshift mutation in *fliN*, and nonsynonymous substitutions in *dppA* (Table 1). These genes encode components of the chemotaxis signaling and flagellar switch complexes that regulate flagellar rotation and directional motility.

**Table 1.** Whole-genome sequencing SNPs information of the “bacterial chemotaxis” pathway genes in M1–M10 mutants.

Gene	Protein ID	Position	Reference	Allele	Protein level variation	locus tag	Products annotation
*cheA*	AFV41613.1	402,336	G	A	Q208*	HP_RS01930	histidine kinase CheA
*fliN*	AFV41803.1	615,121	CA	C	C36fs	HP_RS02875	flagellar motor switch protein
*dppA*	AFV41522.1	315,780	A	G	T66A	HP_RS01470	dipeptide ABC transporter periplasmic dipeptide-binding
		315,786	G	A	G68S		
		316,777	C	A	T398N		
		316,956	T	A	L458I		

† * indicates a stop codon; fs indicates a frameshift mutation.

Although direct functional validation of these mutations was not performed, their convergence within motility- and chemotaxis-related pathways suggests that attenuation of motility may facilitate adaptive biofilm restoration in the Δ*modABD* background under oxidative stress.

## Discussion

**Figure 6.**
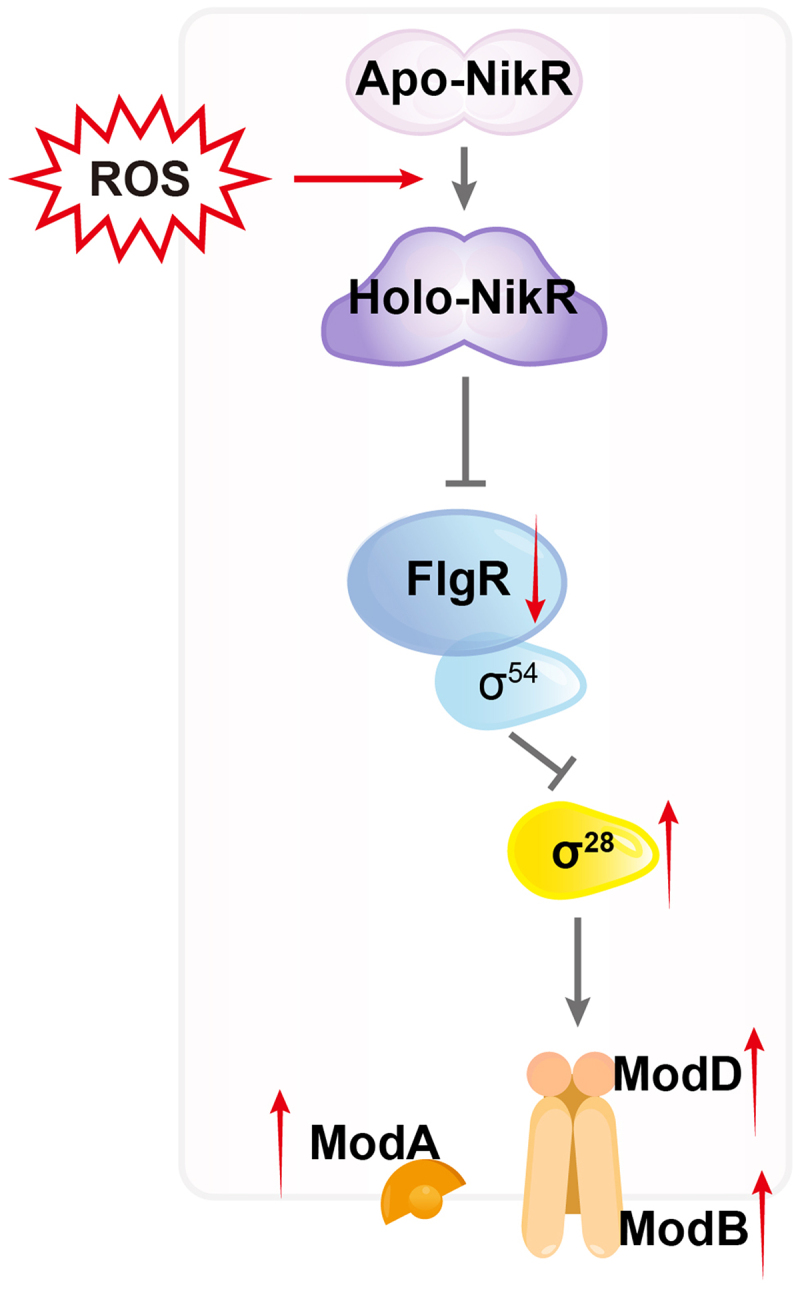
Proposed regulatory mechanism for *H. pylori* biofilm transition. Schematic representation of ROS-mediated biofilm induction. ROS induces a conformational change in NikR from apo to holo form. Holo-NikR represses *flgR* expression. Reduced FlgR levels relieve inhibition of σ^28^-dependent *modABD* transcription, activating molybdate transport. This molecular cascade promotes the transition from planktonic to biofilm state.

Biofilm formation represents a critical survival strategy for *H. pylori*, facilitating immune evasion and resistance to environmental stress [55,56]. However, the regulatory mechanism governing the transition from planktonic to biofilm states in *H. pylori* remains poorly understood. In this study, we identify a previously uncharacterized regulatory mechanism in which the flagellar regulator FlgR suppresses biofilm formation by inhibiting the molybdate transporter ModABD through σ^28^-dependent transcriptional repression. Crucially, ROS induces a conformational activation of the nickel-responsive regulator NikR, which directly binds to the *flgR* promoter to inhibit its expression. This repression relieves FlgR-mediated inhibition of *modABD*, thereby promoting biofilm initiation (Figure 6).

Our results establish FlgR, a key regulator of flagellar biosynthesis, as a negative regulator of biofilm formation. While FlgR is well-recognized for its role in activating σ^54^-dependent class 2 flagellar genes and repressing σ^28^-dependent class 3 genes [32–35], its involvement in biofilm regulation was previously unknown. The observed downregulation of FlgR and structural flagellar genes in biofilms (Figure S2), coupled with the enhanced biofilm formation in Δ*flgR* mutants across multiple strains (Figure 1; Figure 2(C,D)), demonstrates a paradigm shift in FlgR’s function: beyond orchestrating motility, it actively suppresses the transition to a sessile lifestyle. This functional duality is consistent with observations in other pathogens like *Bacillus subtilis*, where genes responsible for flagellar basal body and filament synthesis are downregulated in mature biofilms [57]. Our findings suggest that *H. pylori* employs a similar strategy, repressing motility-related genes via FlgR downregulation to facilitate biofilm establishment. Mechanistically, FlgR-mediated biofilm suppression operates through repression of σ^28^, which directly activates the molybdate transporter operon *modABD* (Figure 3) – a pathway not previously implicated in biofilm regulation in this pathogen. Although *modA*, *modB*, and *modD* are transcribed as part of a single operon, differences in their relative transcript levels detected by qRT-PCR (Figure S5D) likely reflect differential mRNA stability, internal transcriptional signals within the operon, and condition-dependent modulation by FlgR [58]. Nevertheless, the three genes showed consistent upregulation in the Δ*flgR* mutant, supporting their coordinated regulation.

In bacteria, molybdate transporters have been traditionally characterized as cofactor import systems supporting anaerobic respiration and redox processes [59,60]. In this study, we identified an additional function of the ModABD transporter in maintaining robust biofilm architecture in *H. pylori*. Deletion of *modABD* (Δ*modA*, Δ*modB*, Δ*modD*, Δ*modABD*) significantly impaired biofilm integrity and biomass (Figure 2(A,B); Figure S6). This phenotype was conserved across both standard and clinical antibiotic-resistant strains (Figure 2(C,D)), highlighting its biological relevance. Notably, the reduced EPS components observed in Δ*modA* biofilms under oxidative stress (Figure S7) suggest that molybdate transport may contribute to the biosynthesis or stabilization of the biofilm matrix. These deficiencies likely underlie the architectural defects observed, given that EPS components act as scaffolds mediating intercellular adhesion and structural stability [61]. Consistent with this finding, previous studies have shown that ATP-binding cassette (ABC) transporters play crucial roles in EPS production during bacterial biofilm formation. For instance, the YhdX protein of *E. coli* contributes to matrix assembly by transporting L-amino acids [62], and the *lsrA*-encoded LsrABCD complex mediates AI-2 uptake and is significantly upregulated during biofilm growth [63]. Although ABC transporters are structurally and functionally diverse, these examples underscore a broader functional relevance of ABC transporters, including ModABD, in coupling nutrient transport with biofilm matrix biogenesis. Collectively, our findings establish that ModABD-mediated molybdate transport is critical for EPS biosynthesis, a fundamental process in biofilm maturation.

As a microaerophilic pathogen, *H. pylori* faces oxidative stress during host infection (e.g. phagocyte-derived ROS) and environmental transmission (e.g. atmospheric oxygen) [7,8]. ROS can induce conformational changes in redox-sensitive regulators through residue-specific oxidation. For instance, H_2_O_2_ oxidizes two cysteine residues in the bacterial oxidative stress sensor OxyR, forming an intramolecular disulfide bond that activates its DNA-binding function and triggers antioxidant gene expression [64]. Similarly, superoxide anions (O_2_^−^) oxidize the iron-sulfur cluster in SoxR, enhancing its affinity for the *soxS* promoter [65].

Our data reveal that ROS induces a conformational shift in NikR from its apo form to the DNA-binding holo state (Figure 4(E)). In this activated state, NikR directly represses *flgR* expression (Figure 4(A-C)), thereby relieving FlgR-mediated inhibition of *modABD* to promote biofilm formation (Figure 6). Notably, ROS mimic nickel-induced activation of NikR (Figure 4(E)), positioning its role as a multimodal sensor that integrates metal ion and oxidative stress signals. This finding is consistent with NikR’s established function in global stress responses [66], yet represents the first report of its regulatory effect on flagellar gene expression. Moreover, a recent study from our laboratory demonstrated that the host macrophage – derived ROS similarly promote *H. pylori* biofilm formation, and this effect is abolished by the ROS scavenger N-acetylcysteine (NAC) [54]. This mechanism underscores the sophisticated integration of metal homeostasis (Ni^2+^ sensing by NikR) and oxidative stress response into biofilm regulation.

The potential role of motility attenuation in *H. pylori* biofilm formation was further supported by compensatory evolution in Δ*modABD* strains. Upon iterative low-dose H_2_O_2_ exposure (Figure 5(A)), evolved lineages (e.g. M7) evolved to restore biofilm biomass to near-WT levels (Figure 5(B)). Whole-genome sequencing revealed that mutations were enriched in motility and chemotaxis pathways (Figure 5(C); Table 1), including *cheA*, *fliN*, and *dppA*, suggesting that *H. pylori* can reprogram motility-related functions to circumvent ModABD deficiency under oxidative stress – a strategy resembling the motility-biofilm trade-off reported in other pathogens [67–69]. These observations, together with the FlgR–σ^28^–ModABD regulatory pathway described above, indicate that both ModABD transporter and evolutionary adaptation converge on motility attenuation as a mechanism facilitating biofilm commitment. Collectively, our findings highlight FlgR as a key regulator orchestrating the motility-to-biofilm transition in *H. pylori* under oxidative stress.

While this study elucidates how *H. pylori* integrates environmental cues (particularly oxidative stress) through NikR to modulate FlgR activity and control the transition between motile and biofilm lifestyles via the molybdate transporter ModABD (Figure 6), several limitations should be noted. First, the proposed redox activation of NikR requires further validation through cysteine mutagenesis and structural analysis. Second, the adaptive mutations identified in motility-related genes were not functionally characterized. Finally, the potential link between molybdate transport and EPS biosynthesis warrants detailed metabolic and compositional profiling. Despite these limitations, our findings establish a mechanistic framework for understanding how *H. pylori* responds to oxidative stress to regulate biofilm development and may guide future strategies to disrupt biofilm-mediated persistence.

## Supplementary Material

Ethical_Approval.pdf

Supplementary data_Clean_2025Oct.docx

## Date availability

The data that support the findings of this study are openly available in Figshare at https://doi.org/10.6084/m9.figshare.29468219, reference number 29,468,219. The RNA sequencing data have been deposited in the NCBI SRA database under Bioproject accession PRJNA648673 and PRJNA1248242. The genome resequencing data for the H57 and M1–M10 strains are available under PRJNA1052444 and PRJNA1248678, respectively.
