# Supplementary material for: ROS-induced allosteric modulation of NikR promotes Helicobacter pylori biofilm formation by attenuating FlgR-dependent inhibition of the molybdate transport system
Source: Virulence. 2025 Nov 12;16(1):2589562. doi: 10.1080/21505594.2025.2589562 (PMC12622350; doi:10.1080/21505594.2025.2589562)
Supplement: Ethical_Approval.pdf [file KVIR_A_2589562_SM8733.pdf]

山东大学基础医学院伦理委员会

审 批 件

编号： ECSBMSSDU2020-1-021

项目名称：SpoT 调控氧应激诱导幽门螺杆菌形成生物膜分子机制研究

项目负责人：孙允东　　职称：　　教授　　联系电话：15315557925

负责研究单位：山东大学

合作研究单位：

研究起止时间：2021 年 1 月-2024 年 12 月

拟申报项目类别：国家自然科学基金面上项目

审查意见：

经本委员会审查，研究者的资格、经验符合实验要求；研究方案符合《赫尔辛基宣言》以及《涉及人的生物医学研究伦理审查办法》等有关法律法规和伦理规范的要求；知情同意方法适当；受试者可能遭受的风险与研究预期的受益相比适当。

同意开展该项目的研究，但在研究过程中应接受本委员会的监督，研究者在研究结束后，应当向本委员会递交最终报告，包含对于研究发现及研究结论的总结。

山东大学基础医学院伦理委员会

主任委员：

2020 年 3 月 30 日

地址：济南市文化西路 44 号山东大学趵突泉校区院内

联系电话：（+86 531）88382077　　E-mail：lunli82077@163.com

贵州医科大学附属医院医学伦理委员会

伦理审查批件

批件号：　　　　　　　　　　　　　　　　2016伦审第 56 号

项目名称	**幽门螺杆菌感染治疗前后胃内菌群变化及其临床意义**						
项目类别	☐科研课题　A.应用基础研究　B.临床研究　C.药学　D.中西医结合　E.其他<br>☐新技术应用　☐其他（请注明）						
申请科室	基础医学院微生物学教研室	科室负责人	陈峥宏	项目负责人	陈峥宏	起止时间	2017年1月~2019年12月
研究目的	了解幽门螺杆菌（Helicobacter pylori,Hp）感染治疗前后患者胃内菌群变化及胃内耐药非Hp与疾病和疗效的关系						
涉及人体研究内容	对临床胃镜检查疑为Hp感染相关疾病的患者进行胃黏膜样本的采集，分离培养胃黏膜中Hp和非Hp						
可能出现的不良反应与危害和防治与补偿措施	(如空格填写不下，可以附件形式附后)<br>胃镜检查是临床进行胃部疾病诊断的常用手段之一，本项目是在临床进行胃镜检查的患者中筛选研究对象，研究对象接受的胃镜检查和胃黏膜取样是临床诊断的需要，不会因为项目实施造成对患者的伤害。						

申请人（项目负责人）承诺：

以上所填内容属实，如获批准，我将严格遵守《医学伦理委员会章程》，并按照提供的方案进行项目研究。

签字：陈峥宏　　　日期：2016年3月1日

审查途径	快速审查☐	书面审查 ■
	会议审查☐	会议日期：
	其中：同意0票，作必要修改后同意0票，作必要修改后重审0票，不同意0票，暂停已批准的试验0票	
	结论：■同意　☐不同意　☐暂停已批准的试验	

审查意见：

经我院伦理委员会讨论审核，认为该项目符合卫生部《涉及人的生物医学研究伦理审查办法（试行）》及赫尔辛基宣言关于生物人体试验的相关规定，同意在我院收集样本开展本项研究。

主任委员签字：刘健（印）

（盖章）

日期：

附：申请人（项目负责人）简要信息

姓名	陈峥宏	性别	女	学历学位	博士/博士	职称职务	教授
电话	0851-88174015	手机	13985006815	E-mail	chenzhenghong@gmc.edu.cn		
通讯地址	贵阳花溪贵州医科大学微生物学教研室	邮编	550025				
研究方向	细菌的致病性和遗传多样性						

本申请表一式两份，一份交项目负责人保管，一份交医院科研处留存归档
