# Supplementary material for: ROS-induced allosteric modulation of NikR promotes Helicobacter pylori biofilm formation by attenuating FlgR-dependent inhibition of the molybdate transport system
Source: Virulence. 2025 Nov 12;16(1):2589562. doi: 10.1080/21505594.2025.2589562 (PMC12622350; doi:10.1080/21505594.2025.2589562)
Supplement: Supplementary data_Clean_2025Oct.docx [file KVIR_A_2589562_SM8732.docx]

**Supplementary data**

**Figures S1 to S10**

**
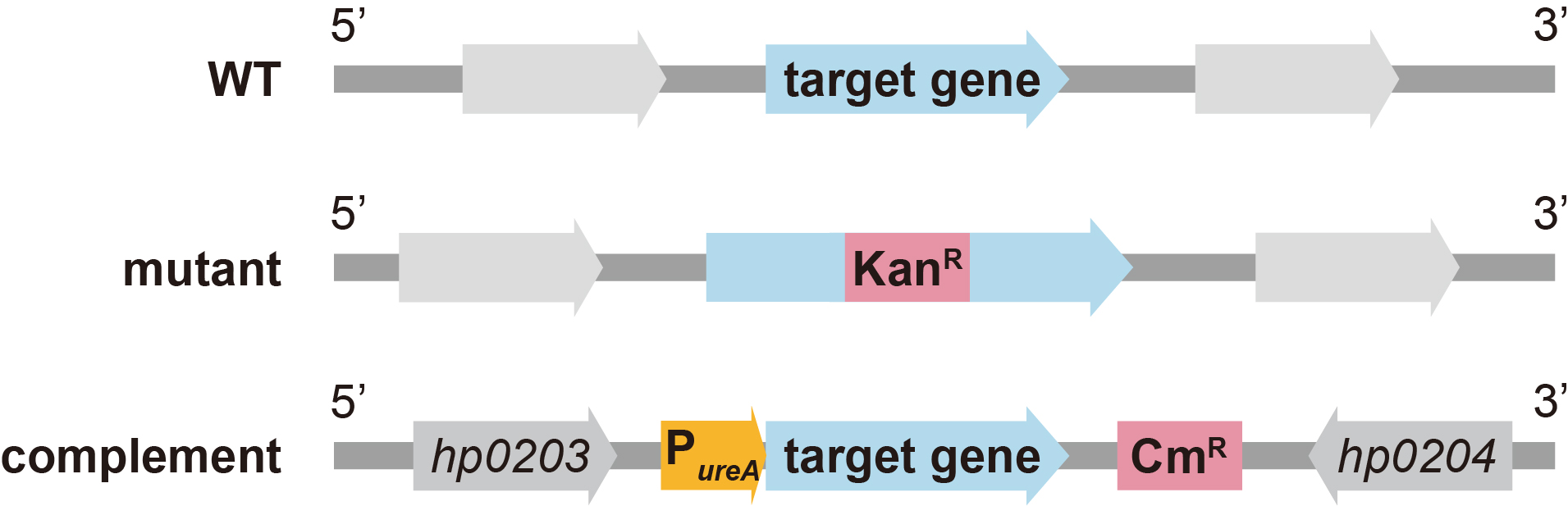
**

**Figure S1.** Schematic of the mutant and complemented strains. Red: resistance cassette; black: *H. pylori* 26695 genomic sequence; blue: target gene; yellow: promoter of the *ureA* gene.


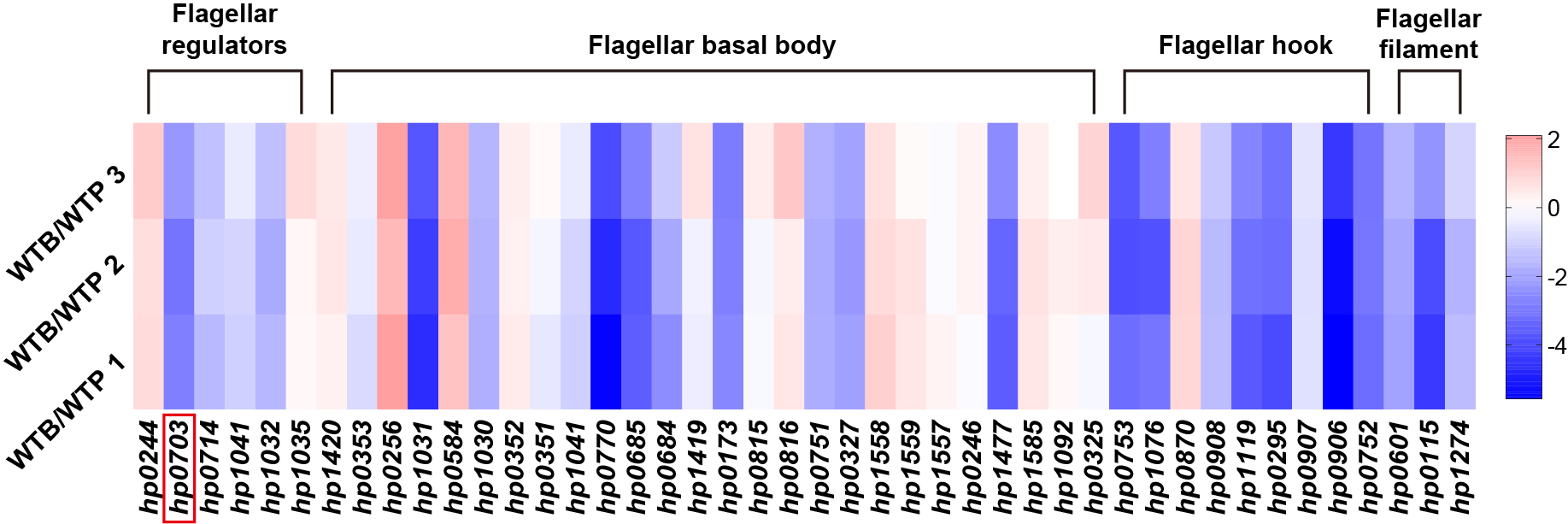


**Figure S2.** Transcriptomic analysis of flagellar assembly genes during *H. pylori* biofilm transition. Heatmap showing the differential expression of flagellar assembly-related genes in WT biofilm cells (WTB) and planktonic cells (WTP). The red box highlights the *flgR* gene (*hp0703*). Expression values are presented as log_2_(fold change).


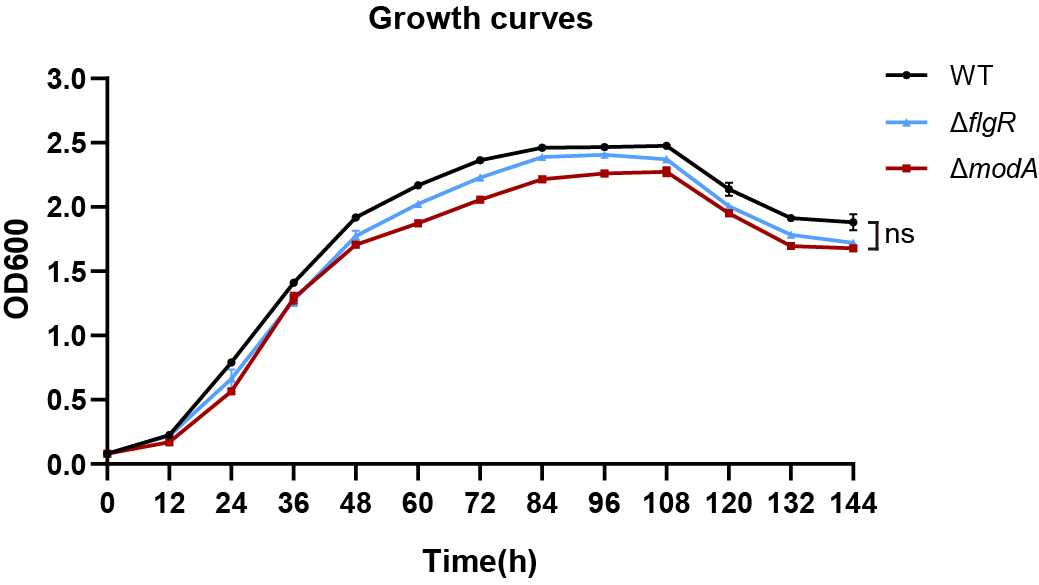


**Figure S3.** Growth curves of WT, Δ *flgR*, and Δ*modA* strains in liquid culture over time. Cultures were initiated at OD_600_ = 0.08 and incubated at 37°C under microaerobic conditions with shaking (120 rpm). The OD_600_ value was measured every 12 h. Data represent mean ± SD of three biological replicates. Ns, no significant.


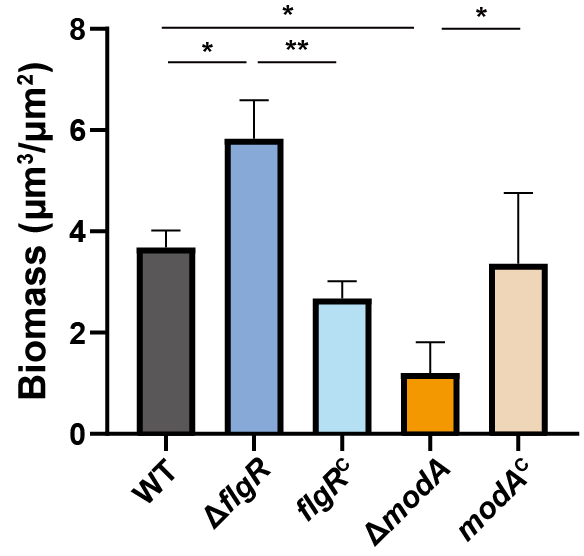


**Figure S4.** Biofilm biomass of the WT, Δ*flgR*, *flgR*^C^, Δ*modA* and *modA*^C^ strains. Quantitative analysis of biofilm biomass (μm^3^/μm^2^) was performed using COMSTAT based on CLSM z-stack images. Data represent the mean ± SD from triplicate measurements. **P* < 0.05, ***P* < 0.01.


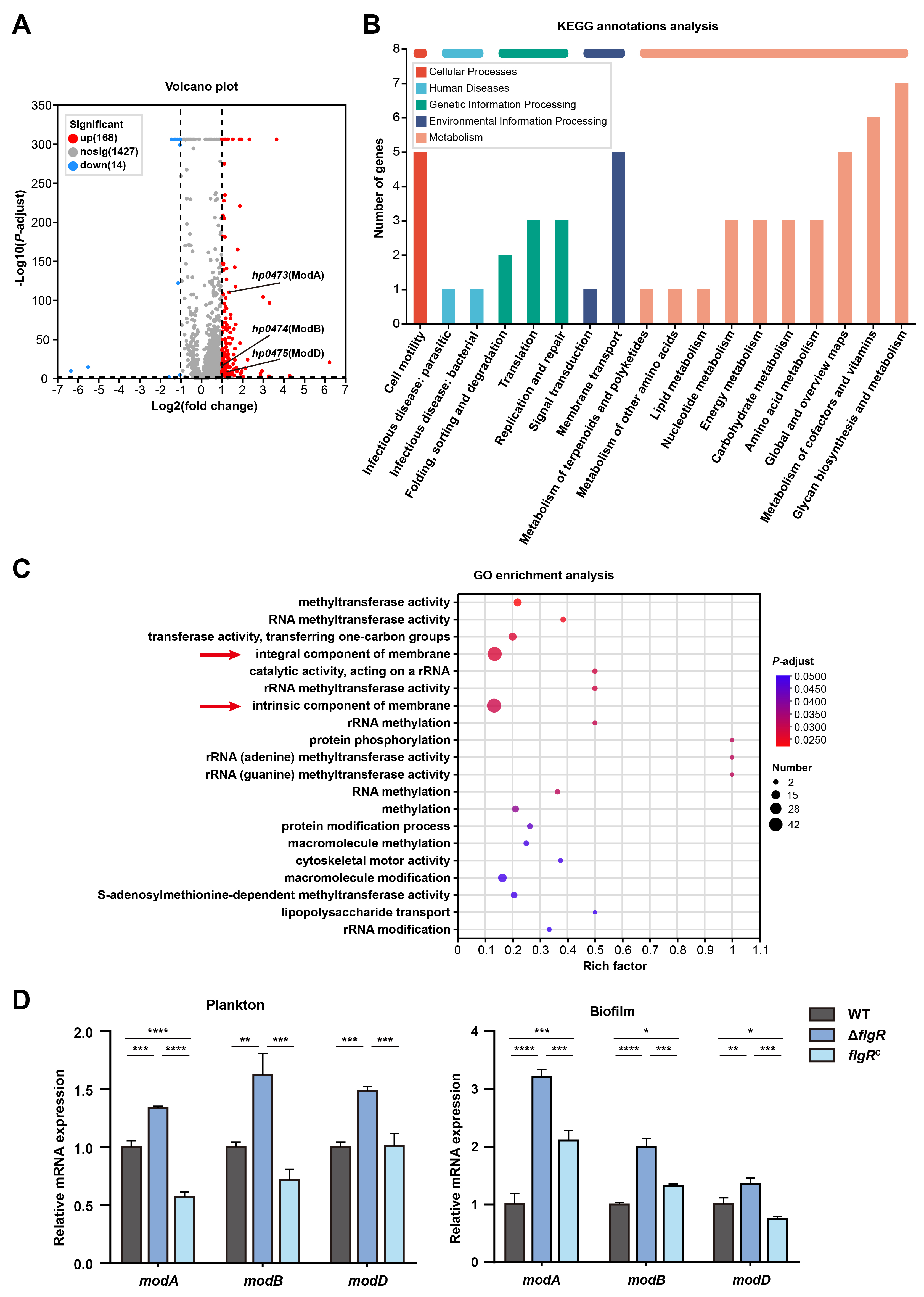


**Figure S5.** Transcriptomic and validation analysis of the Δ*flgR* mutant under oxidative stress.

(A) Volcano plot of DEGs in the Δ*flgR* compared to the WT strain after 1 h of 50 μM H_2_O_2_ treatment. DEGs were identified using DESeq, with an adjusted *P*-value < 0.05 and | log_2_(fold change) | > 1. Red and blue dots indicate significantly upregulated and downregulated genes in the Δ*flgR* mutant, respectively. (B) KEGG pathway annotation of DEGs. Functional categories are indicated in the legend and divided into five major groups: metabolism, genetic information processing, environmental information processing, cellular processes, and human diseases. (C) GO enrichment analysis of DEGs. Rich factor calculated as the ratio of DEGs annotated to a specific GO term to the total number of genes annotated to that term. Dot size reflects the number of genes in each GO term, and dot color corresponds to different adjusted *P*-values. (D) Relative mRNA expression levels of *modA*, *modB*, and *modD* in WT, Δ*flgR*, and *flgR*^C^ strains under planktonic and biofilm conditions, using *16S* *rRNA* as the internal control. Data represent the mean ± SD from triplicate measurements. **P* < 0.05, ***P* < 0.01, ****P* < 0.001, *****P* < 0.0001.


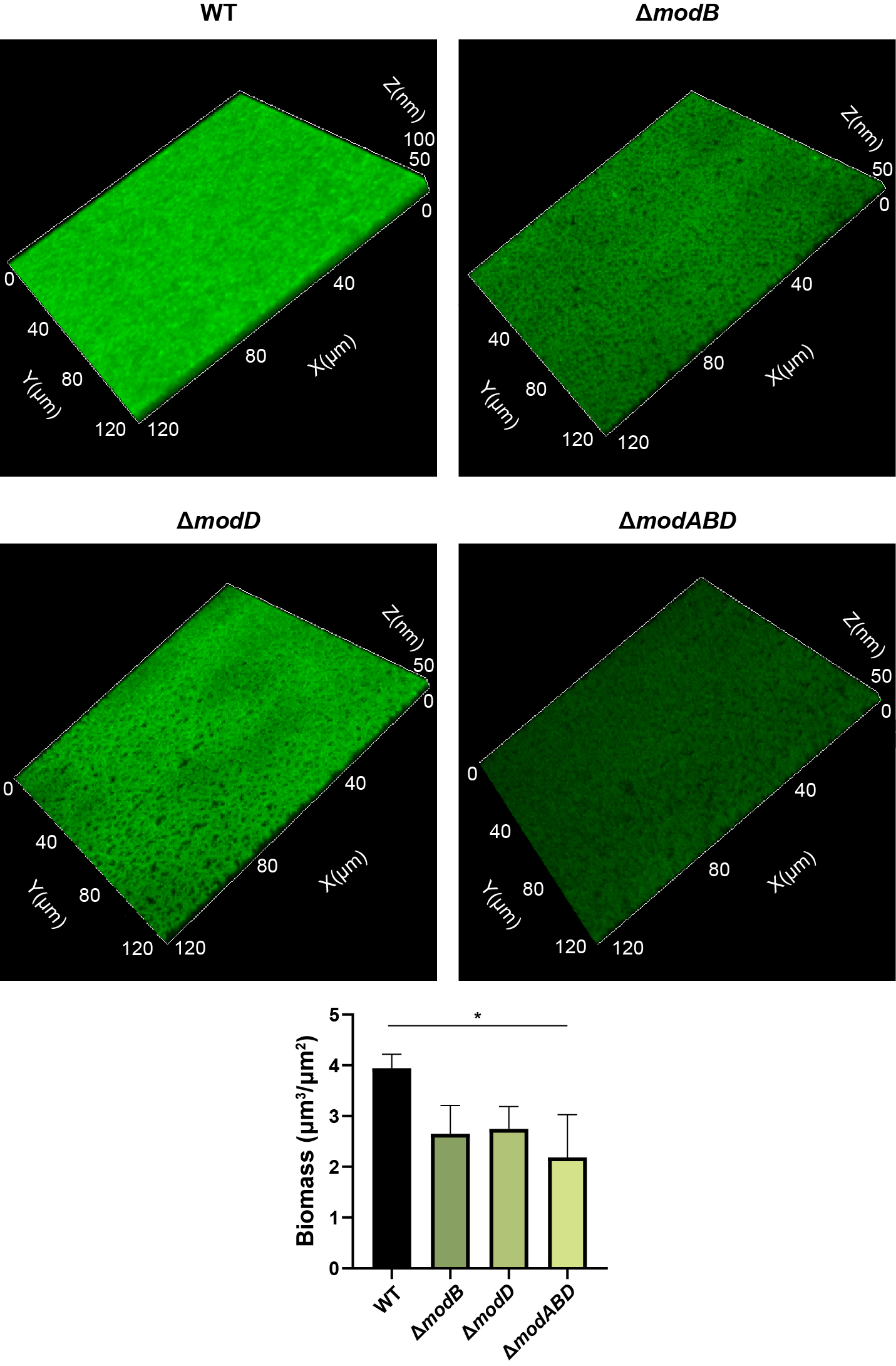


**Figure S6.** CLSM images of biofilms formed by WT, Δ*modB*, Δ*modD*, and Δ*modABD* strains. SYTO 9-stained biofilms formed on medium containing 50 μM H_2_O_2_ after 72 h under microaerobic incubation. Representative images from three independent biological replicates are shown. Quantitative analysis of biofilm biomass (μm^3^/μm^2^) was performed using COMSTAT based on CLSM z-stack images. Data represent the mean ± SD from triplicate measurements. **P* < 0.05.


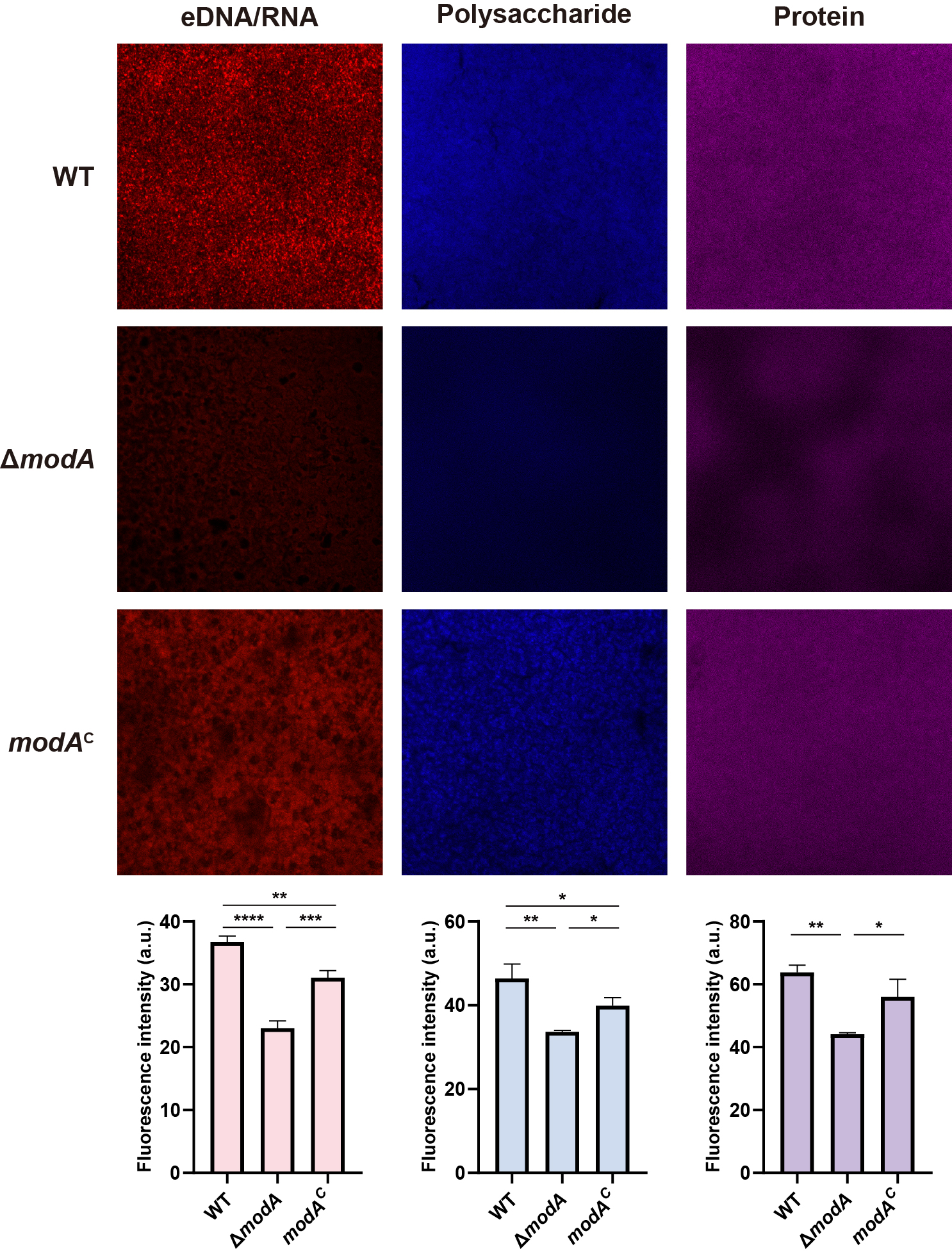


**Figure S7.** Compositional analysis of EPS in *H. pylori* biofilms. CLSM images showing EPS components in biofilms formed by WT, Δ*modA*, and *modA*^C^ strains on medium containing 50 μM H_2_O_2_ after 72 h. Extracellular DNA/RNA (eDNA/RNA), polysaccharides, and proteins in the EPS matrix were stained with PI, Calcofluor White, and SYPRO Ruby biofilm matrix stain, respectively. Representative images from three independent biological replicates are shown. Fluorescence intensity was quantified using ImageJ based on mean gray value from CLSM images. Data represent the mean ± SD from triplicate measurements. **P* < 0.05, ***P* < 0.01, ****P* < 0.001, *****P* < 0.0001.


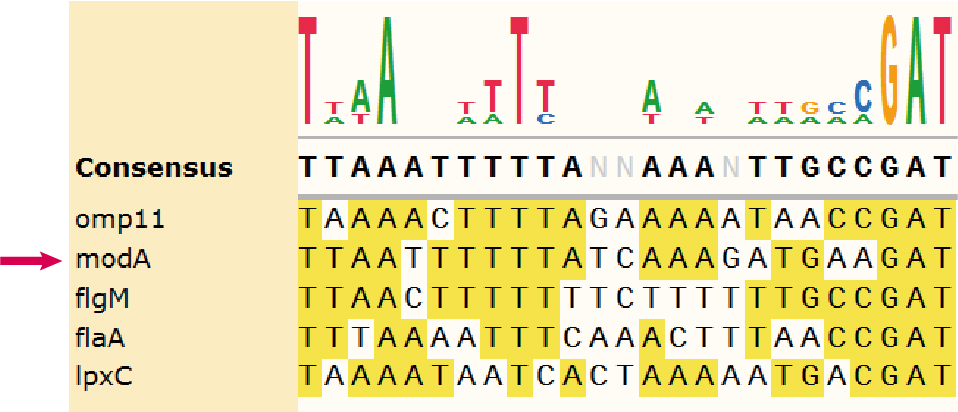


**Figure S8.** A putative σ^28^-binding site in the *modA* promoter. Sequence alignment of the *modA* promoter region with the σ^28^ consensus sequences (5’-TAAANNNNNNNNNNCGAT-3’), performed using ClustalW and visualized with SnapGene software (v4.3.6).


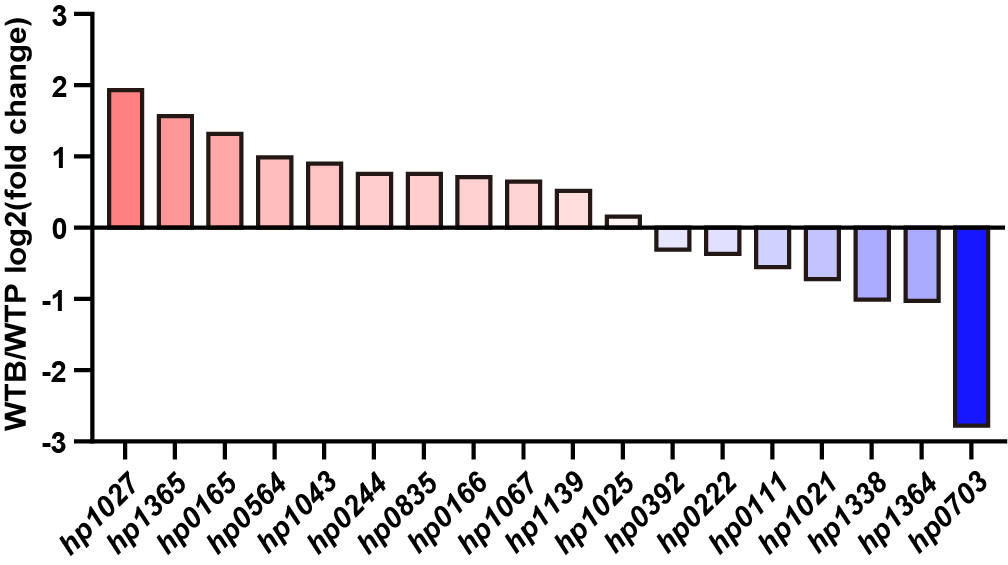


**Figure S9.** Transcriptomic analysis of transcription factor genes during *H. pylori* biofilm transition. Bar chart showing the differential expression of transcription factor genes in WT biofilm cells (WTB) and planktonic cells (WTP). Expression values are presented as log_2_(fold change).


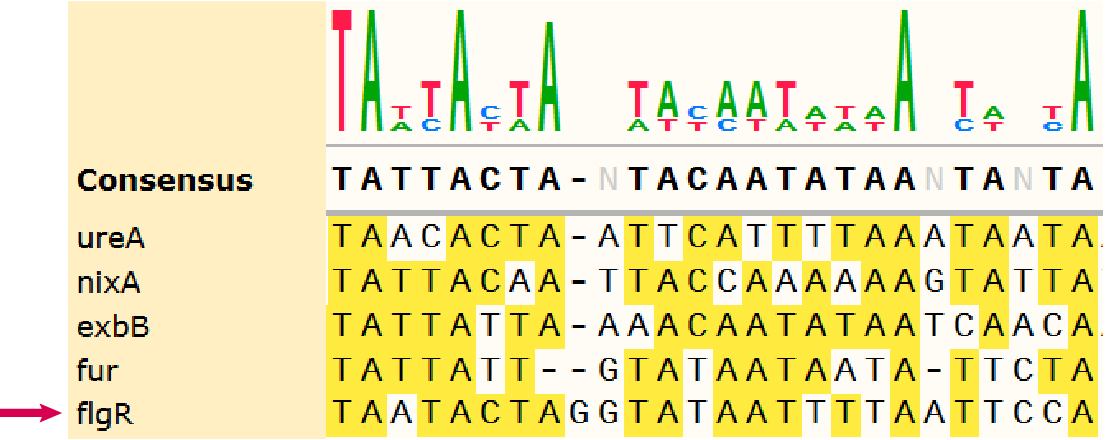


**Figure S10.** A putative NikR-binding site in the *flgR* promoter. Sequence alignment of the *flgR* promoter region with the NikR consensus sequences (5’-TRWYA-N_15_-TRWYA-3’), performed using ClustalW and visualized with SnapGene software (v4.3.6).

**Table**

**Table S1.** Primers and their sequences used in this study. “F” and “R” in each primer name indicate the forward and reverse primers, respectively.

| Primers | Sequences (5’→3’) |
| --- | --- |
| *16S rRNA*-F | GTGTGGGAGAGGTAGGTGGA |
| *16S rRNA*-R | GTTTAGGGCGTGGACTACCA |
| *flgR*-F | CAGGCCTTAAAAGTCGCAAG |
| *flgR*-R | TGAACGCCCCTTTTTGATAC |
| *flgR*-SF | AACTGCAGAGCTCTCAGCCCCCTAGAAG |
| *flgR*-SR | GGAATTCTGGTGGATGAAATGAGCAAA |
| *flgR*-XF | CGGGATCCTATCAAAAAGGGGCGTTCAC |
| *flgR*-XR | CCATCGATTGGAACCCCTAAACTCACCA |
| *flgR*-C-F | CCATCGATATGAAAATCGCCATTGTAGAAG |
| *flgR*-C-R | AACTGCAGCTACCTTTCCAAAAACAAATCT |
| *modA*-F | GAAAAGCCAGCATGGAAGTC |
| *modA*-R | CAAGGATAACGCTCCAAAGC |
| *modB*-F | ACCATGCGTTTGAGCTTTTC |
| *modB*-R | CACGCTTGGGGGTAAAACTA |
| *modD*-F | AAGCGGAAGTTGTGGCTTTA |
| *modD*-R | AAGCCGATTTTTCGTTGTTG |
| *modA*-SF | AACTGCAGTCCAAGTGTTTCCGGCTAAT |
| *modA*-SR | GGAATTCAACCAACACGCCTTTAGCAT |
| *modA*-XF | CGGGATCCGAAAAGCCAGCATGGAAGTC |
| *modA*-XR | CCATCGATAGCGCTTCAGCTTCGTTAAA |
| *modA*-C-F | CCATCGATATGAAAAATACTTTCAAAGCGT |
| *modA*-C-R | AACTGCAGTTAATCCACAATATAGCCGTAT |
| *modB*-SF | AACTGCAGTCAGGCAAACTCTACGCTCA |
| *modB*-SR | GGAATTCCGCTTTTAATGGGGCTTACC |
| *modB*-XF | CGGGATCCTTCCTTGCCCGCTTCTTTA |
| *modB*-XR | CCATCGATTTGGGGTTATGGCTTACCAA |
| *modD*-SF | AACTGCAGCGAGCTTAACAGAAACGCTTG |
| *modD*-SR | GGAATTCCGCATTAAGCGTAACACTTCG |
| *modD*-XF | CGGGATCCGAGCTTTAATCGCAGCCAAG |
| *modD*-XR | CCATCGATTTGGCTCAACGCTCATTATTT |
| G27-*flgR*-SF | AACTGCAGCGTACCGCTTGAAAATCGTT |
| G27-*flgR*-SR | GGAATTCTGGTGGATGAAATGAGCAAA |
| G27-*flgR*-XF | CGGGATCCTATCAAAAAGGGGCGTTCAC |
| G27-*flgR*-XR | CCATCGATATCAAAAATACCCGCCATGA |
| G27-*modA*-SF | AACTGCAGTCCAAGTGTTTCCGGCTAAT |
| G27-*modA*-SR | GGAATTCAACCAACACGCCTTTAGCAT |
| G27-*modA*-XF | CGGGATCCCAAGCGTATCGCTATGGCTAA |
| G27-*modA*-XR | CCATCGATGCGTATTGGTGGGCTTTAGA |
| H57-*flgR*-SF | AACTGCAGTTCGTATCGCTTGAAAATCG |
| H57-*flgR*-SR | GGAATTCTGGTGGATGAAATGAGCAAA |
| H57-*flgR*-XF | CGGGATCCTATCAAAAAGGGGCGTTCAC |
| H57-*flgR*-XR | CCATCGATGATAAGGGCGGGAAAATCAT |
| H57-*modA*-SF | AACTGCAGATACACCCTAGCCCCAAACC |
| H57-*modA*-SR | GGAATTCCAAGGATAACGCTCCAAAGC |
| H57-*modA*-XF | CGGGATCCTTGAACAAGCCTTGATTACCAC |
| H57-*modA*-XR | CCATCGATGCTTTTACCCGCTCCAGATT |
| Probe-*modA* 1-F | AAACAAACCGCCTCAAACTTTTAAAATACA |
| Probe-*modA* 1-R | AATAAAGCGCTTGAAAAAAATACAATTAAAAAGGC |
| Probe-*modA* 2-F | TTTAATTTTTTATCAAAGATGAAGATTTTAGAGTGAAA |
| Probe-*modA* 2-R | AAATGATTGGAATTGGTGATTATACCTATTT |
| Probe-*modA* 3-F | ACTTCGCCAGCCAGTGCCAAACTCG |
| Probe-*modA* 3-R | AAAATCTTCATCTTTGATAAAAAATTAAAACTTTTAGAA |
| OP*_hp0601_*-F | TAAATTTTCTTTATTATAGCCCATTTTCAT |
| OP*_hp0601_*-R | CATTGTTGTAACTCCTTGTTATAAAAAACC |
| G27-*nikR*-SF | AACTGCAGCATCGGATTTTCTCAACAAGC |
| G27-*nikR*-SR | GGAATTCGGCGATTTTGCTCTCATCAT |
| G27-*nikR*-XF | CGGGATCCGAACCAGCGCATGATAGACA |
| G27-*nikR*-XR | CCATCGATTGGCTGAACGCAAATTAAGA |
| H57-*nikR*-SF | AACTGCAGTCAAAGTGGGAATCGCTACA |
| H57-*nikR*-SR | GGAATTCACAAGCACGGCGATTTTACT |
| H57-*nikR*-XF | CGGGATCCTTTTATGCACCACGCACATT |
| H57-*nikR*-XR | CCATCGATATGGCTTTGGTAAGGACAGC |
| G27-*flgR*-F | CAGGCCTTAAAAGTCGCAAG |
| G27-*flgR*-R | TGAACGCCCCTTTTTGATAC |
| H57-*flgR*-F | TTTGCTCATTTCATCCACCA |
| H57-*flgR*-R | TTTTAGGAGCTGTGGCGTCT |
| Probe-*flgR* 1-F | TTGGGTTGATTTAACTCAAGCAAAT |
| Probe-*flgR* 1-R | AGGTGGAATTAAAATTATACCTAGTATTAAATCA |
| Probe-*flgR* 2-F | TAAGCTTTTTTCTTTTTTTTTGATTTAATACTAG |
| Probe-*flgR* 2-R | TCGTGTTTGTTAGGGCAAAAAA |
| OP*_hp0073_*-F | GTATTAAACGCACTTCTAATAACGAT |
| OP*_hp0073_*-R | TAGCAATGTTTTGATTTACTAAGATTAAC |
| OP*_flgR_*-F | GGTACCGATTGCTCAATCCTTTTTATGAAAGAG |
| OP*_flgR_*-R | AGATCTAGCTTTTCCTTGTTAGGTG |
| OP*_modA_*-F | GGTACCTTTAATTTTTTATCAAAGATGAAGATTTTAG |
| OP*_modA_*-R | GAGCTCAAATGATTGGAATTGGTGA |
